# Addressing the double-burden of diabetes and tuberculosis: lessons from Kyrgyzstan

**DOI:** 10.1186/s12992-017-0239-3

**Published:** 2017-03-15

**Authors:** Jolene Skordis-Worrall, Jeff Round, Matthias Arnold, Aida Abdraimova, Baktygul Akkazieva, David Beran

**Affiliations:** 1 UCL Institute for Global Health, London, UK; 2 School of Social and Community Medicine, University of Bristol, Bristol, UK; 3 Munich Center of Health Sciences, LMU München, Munich, Germany; 4 Institute of Health Economics and Health Care Management, Helmholtz Zentrum München, Oberschleißheim, Germany; 5 Health Policy Analysis Centre, Bishkek, Kyrgyzstan; 6 Division of Tropical and Humanitarian Medicine, Geneva University Hospitals and University of Geneva, Geneva, Switzerland

**Keywords:** Tuberculosis, Diabetes, Health systems, Catastrophic spending, Co-morbidity

## Abstract

**Background:**

The incidence of diabetes and tuberculosis co-morbidity is rising, yet little work has been done to understand potential implications for health systems, healthcare providers and individuals. Kyrgyzstan is a priority country for tuberculosis control and has a 5% prevalence of diabetes in adults, with many health system challenges for both conditions.

**Methods:**

Patient exit interviews collected data on demographic and socio-economic characteristics, health spending and care seeking for people with diabetes, tuberculosis and both diabetes and tuberculosis. Qualitative data were collected through semi-structured interviews with healthcare workers involved in diabetes and tuberculosis care, to understand delivery of care and how providers view effectiveness of care.

**Results:**

The experience of co-affected individuals within the health system is different than those just with tuberculosis or diabetes. Co-affected patients do not receive more care and also have different care for their tuberculosis than people with only tuberculosis. Very high levels of catastrophic spending are found among all groups despite these two conditions being included in the Kyrgyz state benefit package especially for medicines.

**Conclusions:**

This study highlights that different patterns of service provision by disease group are found. Although Kyrgyzstan has often been cited as an example in terms of health reforms and developing Primary Health Care, this study highlights the challenge of managing conditions that are viewed as “too complicated” for non-specialists and the impact this has on costs and management of individuals.

## Background

According to the World Health Organization (WHO), the world is on track to meet the target of a 50% mortality reduction of tuberculosis (TB) [1], but new challenges such as diabetes are challenging this progress [2]. With 6% of TB cases worldwide attributable to diabetes [3], there is the need to bridge the management of diabetes and TB from a health system perspective [4]. Looking at diabetes and TB from the population perspective can be seen as a double burden of disease, with the same population being affected by both conditions. However, at an individual level this is an issue of multi-morbidity/co-morbidity and the challenge this represents for the individual and the health system for management [5].

A WHO collaborative framework on diabetes and TB designed to help policy-makers, public health specialists and healthcare workers address the joint burden of diabetes and TB calls for improved collaboration between diabetes and TB programs, as a part of the broader agenda in strengthening health systems [6]. It describes how past successes in coordination and collaboration between TB and HIV/AIDS programs can be used as a model.

The confluence of diabetes and TB is likely to exacerbate global health inequities and possibly also threaten nascent economic growth in these regions. Low and middle-income countries:

- account for the majority of the world’s population [7]
- have the highest burdens of both diabetes and TB [8]
- are likely to experience a rapidly rising incidence of diabetes by 2030 [9]
- have health systems organized for acute rather than chronic care [10]
- have health systems already challenged by double or triple disease burdens [11] and
- face increasing funding pressures, as health spending is not expected to increase faster than in other countries despite the expected increase in disease burden [12]

Given these challenges, there is a need for studies to examine the joint medical costs of managing both conditions. Kyrgyzstan, located in Central Asia, is a former Soviet Republic with a population of 5·4 million. Life-expectancy at birth is 66 years for men and 73 years for women [13] and has been rising steadily rising since 1960 [14]. It is defined by the World Bank as a low income country [15] and is one of the 18 priority countries in the WHO EURO region for TB control [16]. Data from the National TB center shows a national prevalence of 190 per 100,000 in 2013 with a mortality rate of 11 per 100,000 [17]. In Bishkek, the capital city, there is a prevalence of 131.7 per 100,000 population and mortality rate of 18·1 per 100,000 [17]. This is compared to a prevalence of 73 and mortality rate of 5·1 per 100,000 population respectively in the WHO EURO Region [18]. Kyrgyzstan also has the second highest prevalence of multi-drug resistant TB (MDR TB) among new cases in the European region (at 36.6%) and the highest prevalence of MDR TB among previously treated cases (82.0%). Previously treated cases of TB account for 15.7% of all TB cases, a phenomenon that the WHO suggests may be indicative of clinical failures, the need for additional programme management and possible reinfection and/or misclassification [19]. The International Diabetes Federation estimates a 5·0% prevalence of diabetes in those aged 20 to 79 years in Kyrgyzstan, which is expected to increase to 6·6% by 2035 [20]. Previous work has shown a high number of undiagnosed people with diabetes as well as weak capacity to respond to the needs of people with diabetes [21].

Kyrgyzstan thus constitutes an ideal case study for trying to examine the barriers to the joint management of diabetes and TB, without the significant confounding of HIV/AIDS found in other low-income settings. Highly centralized, vertical TB control programs inherited from the Soviet system, such as those in Kyrgyzstan, are difficult to integrate with the general health services because of insufficient funding, sub-optimal allocation of available resources, poorly developed primary health care services and, often, psychological resistance on the part of TB specialists [22]. The aim of this paper is to examine the joint management of diabetes and TB from the demand and supply side of the health system, to highlight challenges in the management of these conditions.

## Methods

**Fig. 1:**
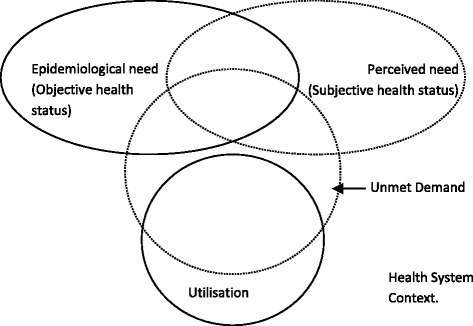
Analytical framework

Ensor and Cooper [23] define demand-side barriers as those that control decisions made by the community, household and individual. Supply-side factors are those that are linked to the provision of healthcare and the health system, and LMICs may struggle with to deliver care in the face of rising costs and cost-containment strategies [24]. The demand for healthcare is influenced by whether or not an individual realizes they are sick and are then “willing and able to seek appropriate healthcare” [23]. This in turn is influenced at the individual and household level by such factors as age, sex, education, income and whether the individual identifies illness and is willing and able to seek appropriate health care. The diagnostic groups and self assessed health status as measured by the multidimensional SF8 index of physical and mental health, will give an indication of need for services [25, 26]. Utilization will be measured using proxy indicators including: Health spending and the number of care-seeking visits in the last 90 days. Using data from the interviews with health service providers will provide the context for what the health system “supplies” to meet the demand of people with either diabetes and/or TB. The analytical framework adopted is presented in Fig. 1.

Quantitative data were collected from individuals adapting a questionnaire used to collect data on TB care seeking in Cape Town, South Africa [27]. The questionnaire was translated from English to Russian and then back-translated to ensure accuracy and coherence and collected the following data:

- Demographic and socio-economic characteristics
- Self reported health status as measured by the SF8, the short form of the SF36 measure of self-reported health
- Direct and indirect, formal and informal treatment costs

The quantitative patient questionnaire was administered at the main facilities in Bishkek where diabetes and TB care would be delivered by trained health systems researchers. Ethical clearance was obtained from University College London (Project 0025/001) and locally. Interview days were randomly selected over a given time period and all individuals attending the clinic on those days were invited to participate in the study.

Quantitative data were analyzed in Stata Version 12. Descriptive statistics are reported using pairwise comparison of means to test for differences. A linear regression model is fit for continuous dependent variables, while logistic regression is used for binary dependent variables. We control for sex, age, level of education and income in all models. Robust standard errors were estimated using bootstrapping to account for skewness in the continuous resource use and cost variables and to adjust for heteroskedacity in the logit models. All significant differences are calculated at the 95% confidence level.

Health expenditure is calculated as the costs of transportation to a health facility, the formal cost of the consultation and all other formal costs occurring at the point of care including diagnostic tests, any unofficial costs paid at the point of care, and any drug costs related to the care seeking event that may not have been incurred at the point of care. For hospital-based care, costs were collected for the most recent overnight admission, observation room and routine outpatient visit occurring during the reference period. Costs were collected for the most recent instance of care seeking only, to reduce recall bias. To measure total expenditure in the last 90 days, these costs were multiplied by the number of times respondents reported visiting that provider in the last 90 days. Catastrophic health care spending is considered, based on the percentage of monthly income spent on medical care. Health spending is described as catastrophic in this study if it equaled or exceeded 10% of total household income [28–30]. This threshold is used as this has been viewed as the level when households are forced to sacrifice other basic needs, sell assets, incur debt or enter poverty [31]. The costs of seeking care outside of a hospital do not include unofficial costs, and only capture the cost of one care seeking episode per non-hospital provider in the last 90 days i.e. respondents were not asked how many times they visited that provider in the last 90 days. As such the costs of non-hospital care seeking are likely to be highly conservative but they are included here for completeness. The SF-8 physical and mental health subscales are used as a measure of health care need.

Qualitative data from health service providers were collected using a tool adapted from the Rapid Assessment Protocol for Insulin Access (RAPIA) in Kyrgyzstan [32]. This required semi-structured interviews with healthcare workers involved in both diabetes and TB care. The aim of these interviews was to identify possible health system barriers and triangulate with quantitative data. Eight healthcare workers were interviewed from facilities identified by local partners as most likely to treat people with diabetes, TB and both conditions. Each interview lasted on average 45 min. Responses were entered into a spreadsheet and translated to English for analysis. In parallel a review of the literature was carried out to provide background information on the situation of diabetes and TB in Kyrgyzstan. The responses from the questionnaires were analyzed thematically focusing on gaining information about TB and joint management of diabetes and TB management building on data already collected and analyzed in a similar way just for diabetes [21, 32].

The qualitative and quantitative data were initially analyzed independently so as to limit any contamination of, or bias in, the results. Once initial findings had been summarized, the quantitative data was used to highlight questions to be asked of the qualitative findings. The aims of those questions were generally to enhance the overall understanding of phenomena observed in the individual data.

## Results

As described in Table 1, 138 (44·7%) of the sample had been diagnosed with either Type 1 or Type 2 diabetes, 139 (45·0%) with TB but not with diabetes, and 32 (10·4%) with both diabetes and TB. Although the total sample was predominantly female (58·3%), co-affected respondents were significantly more likely to be male and better educated on average. Individuals with TB were significantly younger than both other groups and people with diabetes lived in smaller households than did the other two groups. These data are summarized in Table 2, where it is worth noting that despite co-affected individuals appearing to have a much lower household income than the other groups, the wide variance in the income data result in that difference being non-significant.

**Table 1:** Sample size

Illness category	Frequency	Percentage (%)
Co-affected (TB and diabetes)	32	10.4
Diabetes (Type 1 or Type 2)	138	44.7
TB	139	45.0
Total	309	100.0

**Table 2:** Demographic characteristics

	Co-affected	Diabetes	TB affected	Total
	Mean (SD)	Mean (SD)	Mean (SD)	Mean (SD)
Gender Ratio (Male/Female)	1.67 (0.62)	0.42 (0.08)	0.99 (0.17)	0.72 (0.08)
Age	53.91 (10.85)	54.68 (13.17)	30.28 (12.79)	43.62 (17.56)
Education^a^	3.63 (0.56)	3.49 (0.63)	3.26 (0.55)	3.4 (0.60)
Household Size	4.41 (1.88)	3.33 (2.28)	4.13 (1.91)	3.8 (2.12)
Household Income KGS^b^	4941 (4459)	6281 (7524)	8535 (15302)	7156 (11568)

^a^Education is measured as a categorical variable with “1 = no schooling”, “2 = completed primary school”, “3 = completed secondary school” and “4 = completed tertiary education”

^b^KGS = Kyrgyz Som: US$ 1.00 = KGS 46.01 (at average 2011 exchange rate)

TB services in Kyrgyzstan are delivered at all levels of the health system [33]. At the Republican (National) level the TB program is managed by the National Phthisiology Centre, Republican Rehabilitation Centre and the Republican TB Hospital. At Oblasts (Regions) the management of TB is done by TB control centers, hospitals; and finally at the level of the Rayons (Districts) by Rayon and Inter-Rayon TB hospitals and dispensaries. Private providers in Kyrgyzstan are not allowed to deliver care for TB and people with the condition should be referred to a specialized facility. As TB is a large problem in the prison population (prevalence: 1767·0; mortality 430·1 per 100,000) the Department of Corrections under the Ministry of Justice is also a provider of care for TB [33]. Diabetes care is provided at Rayon, Oblast and National level [21, 32]. However, varying patient pathways were found for people with diabetes especially if they required insulin. In looking at the organization of diabetes and TB care as one doctor described it there is a “Lack of integration between the two services”. To highlight this, another doctor from one of the TB specialized facilities described this as “Endocrinologist treats diabetes and we treat TB”. All doctors interviewed highlighted this silo approach or as one doctor described it “parallel” care for people with both diabetes and TB.

Each specialty, diabetes or TB, called the other specialty when needed. Joint consultations were sometimes described, but both conditions were treated in specialist facilities or wards. The choice of admitting someone with diabetes and TB to TB or diabetes wards was based on how “dangerous” the individual is. The way this was viewed was that whichever condition was seen as more serious is prioritized. TB care is provided on an inpatient basis with people being admitted for 2–3 months and then discharged and receiving a further 4 month outpatient treatment. Diabetes care in Bishkek was mainly provided either at Family Medicine Centers (FMC) or through the Endocrinology Dispensary. For diabetes every year one or two inpatient stays at different levels of the health system are planned for patients.

Co-affected individuals should be receiving equivalent care for each of their illnesses, which is not found. The majority of care seeking reported by respondents, took place in a hospital environment. Although the patient survey asked about care seeking from specialists, general practitioners, pharmacists, clinics and other providers outside of the hospital environment, 86.1% of respondents had not sought any form of care outside of a hospital in the preceding 90 days. Of those who did seek care outside of a hospital, 72.1% were people with diabetes (0% co-affected) and 67.4% of those visits were to specialist doctors, while 23.3% were to pharmacists or medicine dispensaries. Individuals with TB only were less likely than people with diabetes only or co-affected individuals to use a hospital drug dispensary or have an outpatient visit, but more likely than individuals with only diabetes to have any contact with the hospital. No differences were observed between groups for emergency care visits (Table 4).

This difference may also be explained by funding for TB. In an analysis of the National Health Accounts, funding for TB in Kyrgyzstan is financed 55% by the state budget and 44% by external donors [33]. The remaining 1% is out of pocket payments. In comparison diabetes does not receive any direct external funding. About US$ 2–3 million spent on diabetes supplies representing 1.4-2.0% of total expenditure on health [32]. Overall 6.5% of total public spending and 18% of external support was spent on TB. Forty percent of all funds allocated for TB in Kyrgyzstan are allocated to inpatient facilities in comparison to 3% for outpatient facilities. The doctors interviewed stated that medicines and care should be free for both conditions. Donors supply medicines for TB, whereas the government does this for diabetes.

All medicines for TB are treatment based on Direct Observed Treatment Short course (DOTS) and DOTS plus programs. and provided through external donors. DOTS is available to all newly diagnosed TB cases throughout Kyrgyzstan and the DOTS plus program is limited to some areas of Kyrgyzstan. Pre-qualified medicines are supplied to Kyrgyzstan mainly through the Global Fund and some government funds. With regards to diabetes poor purchasing practices were found leading to many people needing to purchase some or all of their medicines in the private sector [21]. This was not the case for insulin.

People may not have to pay for their TB medicines, but for other medicines that may be prescribed e.g. vitamins, which are not provided for free. However, financial barriers exist with regards to paying for transportation from FMC to facility where the person is referred to and especially for outpatient management of TB. Others also mentioned administrative issues acting as a barrier with referrals for people with diabetes and TB. Similar challenges we also found for people with diabetes in the previous study [32].

From the questionnaire we find that individuals with only TB are less likely than people with only diabetes to spend anything in order to access care and in particular are less likely to spend on outpatient care or on drugs (Table 3). The overall monthly spend of people with only TB was also less than that of people with only diabetes, as is the monthly spend on drugs. People with diabetes were more likely than co-affected individuals to have spent money on drugs. No other differences were found in the probability of spending on care or in the value of spending on care between the groups (Table 4). People with diabetes spend the most overall and the cost of medicines is the largest portion of total spending for all groups. The differences found in the exit interviews may reflect the fact that people with TB receive their drugs as inpatients and do not have to pay separately for those drugs, or make separate outpatient visits to collect those drugs, whereas this may not be the case for diabetes impacting co-affected individuals.

**Table 3:** Summary of results

	Co-affected	Diabetes	Tuberculosis
Well-being	Mean (SE)	Mean (SE)	Mean (SE)
SF-8 Physical component	35.39 (1.12)	34.43 (0.86)	38.94 (0.95)
SF-8 Mental component	37.41 (1.26)	43.67 (0.74)	41.40 (0.91)
Probability of resource use	Probability (SE)	Probability (SE)	Probability (SE)
Any hospital visit	0.84 (0.07)	0.64 (0.04)	0.89 (0.02)
Any ER/A&E visit	0.13 (0.06)	0.06 (0.02)	0.14 (0.03)
Hospital drug dispensary use	0.81 (0.07)	0.77 (0.04)	0.35 (0.04)
Any outpatient visit	0.94 (0.04)	0.95 (0.02)	0.69 (0.04)
Probability of spending on resource	Probability (SE)	Probability (SE)	Probability (SE)
Any spending	0.44 (0.09)	0.64 (0.04)	0.42 (0.04)
Any drug spend	0.38 (0.09)	0.55 (0.04)	0.33 (0.04)
Any hospital spending	0.09 (0.05)	0.03 (0.01)	0.07 (0.02)
Any outpatient spending	0	0.20 (0.03)	0.08 (0.02)
Probability catastrophic	0.19 (0.07)	0.31 (0.04)	0.08 (0.02)
Total monthly spending (K$)	Mean (SE)	Mean (SE)	Mean (SE)
All monthly spend	837 (305)	996 (193)	424 (77)
Percent of monthly income	21.90 (9.03)	32.16 (8.76)	8.09 (2.28)
Monthly drug spend	613 (213)	509 (76)	207 (38)
Monthly Hospital spending	224 (194)	192 (114)	141 (55)
Monthly outpatient spending	0	295 (135)	75 (30)

**Table 4:** Pairwise comparisons of well-being, service use and spending

	Diabetes v co-affected		Tuberculosis v co-affected	Tuberculosis v diabetes
	Coefficient (95% CI)		Coefficient (95% CI)	Coefficient (95% CI)
Well-being				
SF-8 Physical component	−0.966 (−5.720 to 3.788)		3.548 (−1.203 to 8.299)	4.514 (1.602 to 7.425)***
SF-8 Mental component	6.254 (1.843 to 10.665)**		3.982 (−0.426 to 8.390)	−2.272 (−4.973 to 0.430)
Probability of resource use				
Any hospital visit	−0.177 (−0.361 to 0.006)		0.048 (−0.135 to 0.231)	0.225 (0.113 to 0.338)***
Any ER/A&E visit	−0.067 (−0.205 to 0.071)		0.012 (−0.127 to 0.150)	0.079 (−0.006 to 0.163)
Hospital drug dispensary use	−0.044 (−0.250 to 0.162)		−0.467 (−0.673 to −0.261)***	−0.423 (−0.549 to −0.297)***
Any outpatient visit	0.012 (−0.151 to 0.175)		−0.247 (−0.410 to −0.084)***	−0.259 (−0.359 to −0.159)***
Probability of spending on resource				
Any spending	0.200 (−0.026 to 0.427)		−0.020 (−0.247 to 0.206)	−0.220 (−0.359 to −0.082)**
Any drug spend	0.176 (−0.049 to 0.401)		−0.044 (−0.269 to 0.181)	−0.220 (−0.357 to −0.082)**
Any hospital spending	−0.065 (−0.170 to 0.041)		−0.022 (−0.127 to 0.083)	0.043 (−0.022 to 0.107)
Any outpatient spending	0.196 (0.047 to 0.345)**		0.079 (−0.070 to 0.228)	−0.112 (−0.207 to −0.025)**
Total monthly spending (K$)				
All monthly spend	269 (−236 to 774)		−189 (−694 to 316)	−458 (−768 to −149)**
Monthly drug spend	56 (−165 to 277)		−182 (−403 to 39)	−238 (−374 to −103)***
Monthly Hospital spending	25 (−264 to 313)		−39 (−327 to 249)	−63 (−240 to 113)
Monthly outpatient spending	188 (−131 to 507)		31 (−288 to 350)	−157 (−352 to 39)
Catastrophic spending				
Percent of monthly income	10.62 (−10.56 to 31.81)		−6.50 (−27.73 to 14.74)	−17.12 (−30.47 to −3.77)**
Probability catastrophic	0.121 (−0.060 to 0.303)		−0.113 (−0.295 to 0.069)	−0.234 (−0.349 to −0.120)***

*Significant at 0.05 **Significant at 0.01 ***Significant at 0.001

Access to diagnostic tools for TB was not seen as problematic by the doctors interviewed and this assertion could not be tested with the quantitative data from TB patients. However, the doctors interviewed stated that some people have to pay for their chest x-rays. For diabetes it was found that there were problems with the availability of some laboratory tools and that HbA1c was only available in the private sector [32]. Problems with access to some diagnostic tools for diabetes e.g. blood glucose meters and strips at TB facilities were noted, but basic blood chemistry was available. The quantitative survey asked patients how much they had paid for diabetes test strips. More than a third (35%) of diabetic patients had paid for diabetes test strips, indicating that they had been purchased outside of the public health care system.

As described by one doctor “Narrowly focused specialization complicates management of patients”. This focus on one condition versus another was highlighted through quotes from both a diabetes and TB specialist when they respectively said “We treat diabetes and that’s it” and “Since our main task is TB treatment, the primary treatment is focused on TB. And then we treat the co-morbidity”. All doctors interviewed confirmed this by saying that endocrinologists lacked knowledge of TB and TB specialists lacked knowledge of diabetes. In addition it should be noted that for diabetes it was often said that General Practitioners were not able to treat diabetes and that they were “scared” of treating diabetes, especially using insulin [32]. There has been training in TB management in Kyrgyzstan funded by the Global Fund, but this training focused on TB and HIV/AIDS. Issues around lack of knowledge of TB at lower levels of the health system were mentioned by specialists in that often there were problems identifying TB at FMCs. For diabetes management training had been carried out, but many stated that the challenge was then integrating this into daily clinical practice [32]. These factors may explain why despite requiring “double” care for their diabetes and TB, co-affected individuals actually receive less due to the lack of training and vertical management of the two diseases.

Good TB management is needed for good diabetes management and vice versa. But the challenge, as stated by some of the doctors interviewed, was that people with TB require more food and this is not advisable for people with diabetes. Also TB treatment is focused on inpatient care and “After 2–3 months of treatment, when they complete the intensive treatment phase, start feeling well [they] stop considering themselves as patients.” So the main problem with adherence is when people are discharged from hospital. As people with diabetes and TB need to adhere to two sets of indications and medicines, that may be contradictory, this may impact their use of the health system. In addition cost of treatment may impact adherence and the assessment of catastrophic health payments in the total sample, and comparisons of catastrophic spending between groups found that 20.1% of the total sample spent catastrophically on their healthcare, with households spending an average of 11.8% of total household income on healthcare (Table 3). People with only TB were less likely to incur catastrophic spending than people with only diabetes, while at the same time spent a lower percentage of their monthly income on care (Table 4).

## Discussion

Very different patterns of service utilization by disease group are found. Individuals with TB receive significantly more inpatient care and less outpatient care than the other two groups. People with diabetes have significantly more outpatient visits than people with TB. Co-affected individuals have the most visits to health centers to collect medication, followed by people with diabetes and people with TB. This pattern of service use, when compared with health need, raises two important questions with respect to the sufficiency, equity and efficiency of care. Firstly, co-affected individuals have significantly worse mental health scores than the other two groups and suffer from two diseases, but they neither receive more inpatient care nor more outpatient care than the other two groups. Furthermore, the doctors responding mentioned nothing about additional services for individuals co-affected by both diseases. This suggests that care for people affected by both diseases may be under-provided. Secondly, co-affected individuals have TB, but do not appear to receive the same inpatient-based treatment for their TB as do people affected by TB only. If inpatient care for TB is the more effective form of care in this context, then co-affected individuals are being treated with less effective care and this would be inequitable. If however, outpatient care is as effective as inpatient care in this context, then it is technically inefficient to provide inpatient-based care to individuals affected by TB alone. This highlights the challenge emphasized by Riza et al. [34] of what models of care are needed for the co-management of diabetes and TB. Although medicines and care for both conditions should be free, as they are covered by the State Guaranteed Benefit Package, our results show significant spending on drugs and care, often at catastrophic levels – co-payments often occur in LMIC even where treatment should be provided free at the point of access [35]. That said it is worrying to see the low levels of spending by co-affected individuals, as it would be expected they would spend more than individuals with only one condition.

The analytical framework outlined in Section 1 is simplistic but assisted in linking the concepts of need, service use and service provision in order to reflect on the sufficiency, equity and efficiency of care for individuals co-affected by diabetes and TB. This study has a few limitations in terms of different aspects of its methodology and findings. The interviews on the possible health system barriers were only carried out with 8 doctors and focused on the experience of individuals in Bishkek and not all of Kyrgyzstan. The tool used was an adaptation of an existing tool used for diabetes and may not have been able to capture all aspects of TB management. Parts of the results rely on published studies to fill in certain gaps versus having collected this information first hand. This study claims to provide insight into unmet need for care, but collecting data from patient exit interviews will bias the data towards people who are able to access some form of care. In addition, the very small sample of co-affected individuals has resulted in wide confidence intervals around measure of household income and health spending particularly. This may have resulted in large differences in income and expenditure showing as not statistically significant. That said, the use of multiple measures of need and multiple measures of care use, do indicate robustly, that co-affected individuals are receiving less care than people with lower or equivalent need. Despite these limitations to the authors’ knowledge this is the first study of its kind to look at financial and health system barriers for the co-management of diabetes and TB.

## Conclusion

Kyrgyzstan has been cited as an example in terms of health reforms and developing Primary Health Care [36], however this study highlights the limits of this success in managing conditions that are viewed as “complicated” thus requiring specialist management. Verticalization of the health system impacts patient outcomes as well as health system targets for TB control. In many countries such as Kyrgyzstan, the link between TB and HIV is smaller than diabetes and TB [37, 38] and this will require a shift in a variety of health system factors including: healthcare provision and treatment guidelines; organization of the health system; government priorities and donor perspectives. This study shows that the WHO Collaborative Framework for Care and Control of Tuberculosis and Diabetes are in need of implementation at country level especially with regards to their aim of strengthening collaboration between diabetes and TB services [4]. Although a lot of emphasis in this framework is put on case detection, there is the need to guarantee quality management of diabetes and TB to ensure that the focus of the health system’s response is not the management of the individual conditions, but the management of the individual.

## Abbreviations

AIDS: Acquired immune deficiency syndrome
DOTS: Direct observed treatment short course
HIV: Human immunodeficiency virus
TB: Tuberculosis
WHO: World Health Organisation

## References

[1] WHO. MDG 6: combat HIV/AIDS, malaria and other diseases. 2014. http://www.who.int/topics/millennium_development_goals/diseases/en/ (accessed 30 Oct 2014).

[2] Pan SC, Ku CC, Kao D, Ezzati M, Fang CT, Lin HH (2015). Effect of diabetes on tuberculosis control in 13 countries with high tuberculosis: a modelling study. Lancet Diabetes Endocrinol.

[3] Lönnroth K, Jaramillo E, Williams BG, Dye C, Raviglione M (2009). Drivers of tuberculosis epidemics: the role of risk factors and social determinants. Soc Sci Med.

[4] Stop TB Department and Department of Chronic Diseases and Health Promotion World Health Organization (2011). The international union against tuberculosis and lung disease. Collaborative framework for care and control of tuberculosis and diabetes.

[5] Beran D, Sartorius N, Holt RIG, Maj M (2015). Difficulties facing the provision of care for multi-morbidity in low-income countries. Comorbidity of mental and physical disorders Key issues ment health.

[6] Lonnroth K, Roglic G, Harries AD (2014). Improving tuberculosis prevention and care through addressing the global diabetes epidemic: from evidence to policy and practice. Lancet Diabetes Endocrinol.

[7] World Bank. Data. 2013. http://data.worldbank.org/indicator/SP.POP.TOTL (accessed 30 Oct 2013).

[8] Restrepo BI (2007). Convergence of the tuberculosis and diabetes epidemics: renewal of old acquaintances. Clin Infect Dis.

[9] IDF (2011). International diabetes federation diabetes atlas.

[10] WHO (2002). Innovative care for chronic conditions: building blocks for action.

[11] Salomon JA, Vos T, Hogan DR (2012). Common values in assessing health outcomes from disease and injury: disability weights measurement study for the Global Burden of Disease Study 2010. Lancet.

[12] Dieleman JL, Templin T, Sadat N (2016). National spending on health by source for 184 countries between 2013 and 2040. Lancet.

[13] WHO. Countries - Kyrgyzstan. 2015. http://www.who.int/countries/kgz/en/ (accessed 12 May 2015).

[14] World Bank (2016). Life exepctancy at birth, total (years).

[15] World Bank (2013). Kyrgyz Republic.

[16] Europe WHO (2007). Epidemiology of tuberculosis in Europe.

[17] Akkazieva B, Temirov A, Atun R (2008). Methodological Guideline: NHA Sub-accounts for TB system in Kyrgyzstan.

[18] WHO. Kyrgyzstan Country Health Profile - Tuberculosis. 2015. http://www.who.int/countries/kgz/en/ (accessed 30 June 2015).

[19] European Centre for Disease Prevention and Control/WHO Regional Office for Europe (2012). Tuberculosis surveillance and monitoring in Europe 2012.

[20] IDF (2013). IDF Diabetes Atlas.

[21] Beran D, Abdraimova A, Akkazieva B, McKee M, Balabanova D, Yudkin JS (2013). Diabetes in Kyrgyzstan: changes between 2002 and 2009. Int J Health Plann Manage.

[22] WHO Europe (2007). Tuberculosis and health systems.

[23] Ensor T, Cooper S (2004). Overcoming barriers to health service access: influencing the demand side. Health Policy Plan.

[24] Jakovljevic M, Vukovic M, Chen CC (2016). Do health reforms impact cost consciousness of health care professionals? results from a nation-wide survey in the Balkans. Balkan Med J.

[25] Turner-Bowker DM, Bayliss MS, Ware JE, Kosinski M (2003). Usefulness of the SF-8 Health Survey for comparing the impact of migraine and other conditions. Qual Life Res.

[26] Ware JE (2000). SF-36 health survey update. Spine.

[27] Skordis-Worrall J, Hanson K, Mills A (2011). Estimating the demand for health services in four poor districts of Cape Town, South Africa. International health.

[28] Pradhan M, Prescott N (2002). Social risk management options for medical care in Indonesia. Health Econ.

[29] Ranson MK (2002). Reduction of catastrophic health care expenditures by a community-based health insurance scheme in Gujarat, India: current experiences and challenges. Bull World Health Organ.

[30] Wagstaff A, van Doorslaer E (2003). Catastrophe and impoverishment in paying for health care: with applications to Vietnam 1993–1998. Health Econ.

[31] Russell S (2004). The economic burden of illness for households in developing countries: a review of studies focusing on malaria, tuberculosis, and human immunodeficiency virus/acquired immunodeficiency syndrome. Am J Trop Med Hyg.

[32] Abdraimova A, Beran D (2009). Report on the rapid assessment protocol for insulin access in Kyrgyzstan.

[33] Akkazieva B, Temirov A, Timoshkin A, Atun R (2009). Review of total health expenditures on TB programme in Kyrgyzstan, 2007: NHA Sub-accounts on TB control programme.

[34] Riza AL, Pearson F, Ugarte-Gil C (2014). Clinical management of concurrent diabetes and tuberculosis and the implications for patient services. Lancet Diabetes Endocrinol.

[35] Wang Q, Fu AZ, Brenner S, Kalmus O, Banda HT, De Allegri M (2015). Out-of-pocket expenditure on chronic non-communicable diseases in sub-Saharan Africa: the case of rural Malawi. PLoS One.

[36] Balabanova D, McKee M, Mills A (2011). Good health at low cost’ 25 years on. What makes a successful health system?.

[37] Creswell J, Raviglione M, Ottmani S (2011). Tuberculosis and noncommunicable diseases: neglected links and missed opportunities. Eur Respir J.

[38] Marais BJ, Lonnroth K, Lawn SD (2013). Tuberculosis comorbidity with communicable and non-communicable diseases: integrating health services and control efforts. Lancet Infect Dis.

